# Guidelines for student accoucheurs' acceptance in maternal healthcare

**DOI:** 10.1108/IJHCQA-08-2018-0210

**Published:** 2021-02-02

**Authors:** Siphiwe Themba Madlala, Maureen Nokuthula Sibiya, Thembelihle Sylvia Patience Ngxongo

**Affiliations:** 1 Nursing Science, Faculty of Science and Agriculture, University of Zululand, KwaDlangezwa, South Africa; 2 Faculty of Health Sciences, Durban University of Technology, Ritson Campus, Durban, South Africa; 3 Nursing Science, Durban University of Technology, Ritson Campus, Durban, South Africa

**Keywords:** Guidelines for student accoucheurs, Maternal healthcare, Midwifery nursing, Pregnant women, Quality training

## Abstract

**Purpose:**

The quality of maternal healthcare training is the most optimal degree of health in the delivery of effective, efficient and quality healthcare in midwifery discipline. Student accoucheurs studying at the Free State School of Nursing are faced with resistance, discrimination, rejection and unacceptability by pregnant women during their clinical placement at the Free State maternal healthcare institutions. This results in poor quality of training of student accoucheurs in maternal healthcare. Considerable studies have been conducted on males in midwifery nursing, but no guidelines have been developed to facilitate student accoucheurs' acceptance and improvement of the quality of training in maternal healthcare, hence the purpose of this study.

**Design/methodology/approach:**

A descriptive, explorative qualitative design was used in this study. Qualitative focused group discussions ( *n* = 32) were conducted through purposeful sampling method. Data was analysed thematically.

**Findings:**

Three main categories emerged: student accoucheurs' related factors with social interactions and relations as a theme; maternal healthcare users’ related factors with transcultural diversity and socio-economic status as themes; nurse training institutions and maternal healthcare institutions service providers-related factors with gender inequality in the work place as a theme. Ultimately, the guidelines to facilitate acceptance and improvement of quality training of student accoucheurs in maternal healthcare institutions were developed and recommended for implementation.

**Originality/value:**

The paper developed guidelines to facilitate acceptance and improvement of quality training of student accoucheurs in Free State maternal healthcare institutions.

## Introduction

The quality of education and training of nurses, midwives and accoucheurs are low and poor in many countries including South Africa, resulting in a health workforce ill-prepared to effectively respond to rapidly changing and complex existing, and future health systems including maternal healthcare challenges. The training of males in nursing specifically in midwifery has met various challenges worldwide. Sibiya *et al.* (2019) revealed challenges including resistance, discrimination and rejection in the provision of antenatal care to pregnant women during their visits, deliveries and post-natal care in the maternal healthcare institutions. These challenges faced by student accoucheurs are not only present in South Africa but also worldwide Sibiya *et al.* (2019) . Historically, midwifery is an ancient profession exclusively known as a domain for women. Chinkhata and Langley (2018) support the statement by stating that nursing has evolved as a feminine profession despite some men having performed caring roles since the profession's infancy, hence with the reforms brought about by the founder of modern nursing, Florence Nightingale, men were secluded from being trained as nurses especially midwifery nursing. Women have helped each other in childbirth over the years until men stated to enter into nursing profession. Following the Second World War, a chronic shortage of nurses ensued, secondary to the expansion of alternative work opportunities for women and the growth of the general hospital sector ( Chinkhata and Langley, 2018 ). This led to the formal acceptance of the education and training and registration of male nurses.

Over time, however, nursing and midwifery evolved to include males, men have been prominent in the nursing profession during the Middle Ages and Renaissance, the modern image of an “ideal nurse” is feminized. This is primarily because of the Nightingale system for nurse training, wartime and economic factors ( Yang *et al.* , 2004 ). Recently, the numbers of males entering the nursing profession are steadily increasing worldwide, as a consequence of the move to a more gender-balanced profession. Similarly, the present disproportion of males practicing as nurses in Canada also remains a challenge in the new millennium ( Bartfay and Bartfay, 2017 ). The authors revealed that currently there are 270,724 Registered Nurses in Canada, of which 252,763 (93.4%) are female and only 17,961 (6.6%) are males ( Bartfay and Bartfay, 2017 ). While, the latest statistics show just 11% of nurses in the United Kingdom are male, showing that nursing is still a largely gendered profession ( Radford, 2019 ). In Iran and Jordan, men represent 23% and 38% of registered nurses respectively ( Momani, 2017 ). Whereas in the Kingdom of Saudi Arabia, as in many other countries, the nursing profession remains a female-dominated profession, and male nurses represent 18% of all registered nurses in Saudi Arabia's public hospitals, yet only 3% are Saudis ( Momani, 2017 ). According to the United States of America Census Bureau (2013), nearly 350,000 men are employed as nurses therefore it would be fair to conclude that few men practice as accoucheurs in midwifery nursing units. Despite the global increase in the number of males entering the nursing profession, accoucheurs remain a minority group ( Eswi and Saed, 2011 ).

Midwifery, however, may be one of the most exclusively and disproportionately female specialties in the field of nursing, it is time to acknowledge the presence of accoucheurs, the challenges they face and the positive attributes they bring to the profession ( Pilkenton and Schorn, 2008 ). This can only be achieved through the provision of quality midwifery training to student accoucheurs at the training institutions and maternal healthcare institutions where they are placed for clinical practice.

In the eighteenth century, childbirth in most parts of the world remained informal and exclusively managed by women, and in the nineteenth century respectively, midwifery began to change based on the revised curriculum which allowed men to enter into maternal healthcare discipline ( Sellers *et al.* , 2012 ). Hence the current training of accoucheurs exists in this discipline across the globe. This curriculum was not revised to ensure that the training of student accoucheurs was accommodated across all spheres to ensure quality production of accoucheurs. In 1997, South Africa saw the first admission of student accoucheurs in maternal healthcare training institutions under Regulation (R.425) as amended to be trained with female student midwives irrespective of their gender ( Sellers, 1997 ). R.425 gives birth to the first cohort of student accoucheurs and steadily the numbers of males enrolling in midwifery started to increase gradually. These cohorts of student accoucheurs were trained under the same curriculum design for female student midwives with little support to their ongoing daily challenges faced in maternal healthcare institutions where they are placed for clinical practice. Golden (2018) alluded that nursing training institutions should design the curriculum in a way that involves and encourages male student nurses to become comfortable with intimate female care rendered in maternity units.

In South Africa, chapter 2 of the Constitution stipulates the upholding of gender equality at all times. Hence, the percentage of student accoucheurs training in midwifery has steadily risen and continues to do so in the country. Although the numbers of men who choose nursing midwifery as a profession have increased significantly, it remains difficult for men to practice in this area of nursing despite being trained as accoucheurs. Eswi and Saed (2011) agree that though there is an increase in the number of men entering the nursing profession, they remain a minority in the female-dominated profession. The quality of student accoucheurs' training remains disputable in most maternal healthcare institutions due to various factors including gender diversity and inequality.

Gender diversity and inequality occurring in the nurse training institutions and clinical workplace contributes towards the phenomena. Segregation, rejection and discrimination projected by pregnant women towards student accoucheurs during their clinical placement in maternal healthcare institutions have a negative impact towards goal achievement to become accoucheurs ( Sibiya *et al.* , 2019 ). Although some student accoucheurs manage to complete their training, it is not clear if they have gathered enough knowledge and skills to practice as accoucheurs in rendering quality maternal healthcare services to women based on challenges they faced during their clinical placement in maternal healthcare institutions. Moreover, most male student nurses continue to report episodes of exclusion from gender-specific areas of care such as midwifery from either their female counterparts or from the patients ( Golden, 2018 ). Similarly, in countries such as Saudi Arabia, academic institutions and healthcare facilities are required to provide separate gender-based placements in compliance with the cultural values and Islamic religious views of Saudi Arabia resulting in limited male–female interactions in the clinical setting ( Momani, 2017 ). Furthermore, male nursing students typically complete their clinical training on male wards and do not receive training in obstetrics, gynaecology or paediatrics ( Momani, 2017 ).

According to the World Health Organization (WHO), 800 women die per day globally owing to pregnancy-related complications ( Uzabakiriho and Maswime, 2019 ). While South Africa has unacceptably high rates of maternal mortality with a ratio of 189 deaths per 100,000 live births between 2009 and 2016 ( Pilane and Malan, 2018 ). Statistics revealed that the number of women who dies during and after giving birth has increased drastically since 2000 ( Amnesty International, 2014 ). The high maternal mortality rates could be attributed by various contributing factors including shortage of midwives, quality of maternal healthcare service delivery, quality of midwifery training and poor attendance of antenatal care (ANC) visits by pregnant women. In view of these contributing factors, the Department of Health has developed a number of policies and general guidelines pertaining to the care of mother and child to improve maternal healthcare service delivery. Among the developed policies, procedures and general guidelines available in the training institutions include maternal healthcare institutions, there are no existing guidelines available that address the challenges faced by student accoucheurs in maternity units, nor the developed guidelines to ensure quality training of student accoucheurs in midwifery discipline. The worldviews on Evidence-Based Nursing (2015) states that guidelines are important tools in evidence-based practice that can reduce healthcare variation and improve patient outcomes. However, guidelines produced from multiple sources often conflict with one another, which can be confusing for the healthcare practitioner in clinical and training institutions.

The nurse training institutions are constantly producing registered midwives/accoucheurs on an annually basis to enter the working force in a healthcare sector. According to the policy on gender in Malawi, an equal number of males and females should enter tertiary education. However, this is not the case with nursing due to the historical background of nursing being “feminine,” and the challenges men face in nursing, impact negatively on those intending to enter the profession ( Chinkata and Langley, 2018 ). Predominantly, it is notable that the throughput percentages of student accoucheurs become even more lesser than the percentage of their female student midwives' counterparts. According to the South African Nursing Council (SANC) statistics, in 2012 the Free State School of Nursing registered 72 student accoucheurs in a four-year comprehensive course leading to a Diploma in General Nurse (Community, Psychiatry) and Midwife, but only 35 student accoucheurs successfully completed at the end of 2015, while 125 out of 171 female student midwives completed their training respectively ( SANC, 1985 ). Although there is a relatively significant number of student accoucheurs who completed the programme, their readiness and willingness to practice as accoucheurs in maternal healthcare institutions remain unclear.

It is evident that student accoucheurs are faced with difficulties during their training specifically in midwifery discipline. These difficulties maybe contributed by rejection, segregation, discrimination by pregnant women based on their gender including lack of support from their lectures and midwives in maternity units among other factors contribute to these difficulties. This was confirmed by Mthombeni and Phaladi-Digamela (2015) who stated that male learners in nursing in the United States of America have more difficulties than females during their placement in the maternity units. For example, male student nurses are often rejected by patients or patients' families and are not allowed to practice in certain maternity clinical areas such as the labour wards ( Mthombeni and Phaladi-Digamela, 2015 ). In the same vein, Chinkata and Langley (2018) agree that male students in nursing experience various challenges such as isolation, role strain and lack of male role models and that there is a need to provide support and counselling during the course of education and beyond. The minority of student accoucheurs who successfully completed their training may result in the addition of poor maternal healthcare faced by the country if placed in maternal healthcare institutions due to poor quality of training received during their training. Consequently, the study intends to develop guidelines to facilitate student accoucheurs' acceptance in clinical practice with the idealisation of achieving quality trained accoucheurs to capture the pandemic shortage of midwives and unacceptably high rates of maternal mortality in South Africa.

## Method

A descriptive explorative design was used to explore the views of student accoucheurs' experiences encountered during their third and fourth levels of training in maternal healthcare institutions. The main focus of this study was on the acceptance of student accoucheurs by pregnant women during their clinical placement in the maternity units. Therefore, a descriptive explorative design was chosen to invent the full nature of the phenomena, the way it is manifested and establishing the clear picture of the situation as it is naturally happening without manipulation of any variables ( Polit and Beck, 2014 ; Schmidt and Brown, 2009 ). The informants were selected during their fourth year level of clinical placement in post-natal units in the maternal healthcare institutions at accredited by the South African Nursing Council for student nurse training purpose in the Free State province. The participants were purposefully selected based on the aim of the study which was on the development of guidelines to support student accoucheurs acceptance by pregnant women during their clinical training in midwifery discipline. The development of the guidelines to support student accoucheurs during their placement in maternity units was based on the findings from the student accoucheurs experiences regarding their acceptance by pregnant women during their clinical placement in maternity units.

### Sampling and data collection

The total of 32 student accoucheurs between the ages of 20 and 24 years studying in the Free State School of Nursing were purposively selected to be included in the study. Purposive sampling is a method often used when the researcher wants a sample of experts ( Polit and Beck, 2012 ). Furthermore, 10 out of the entire 33 maternal healthcare institutions accredited by SANC for midwifery training in the Free State province were selected as study sites. All this study sites are situated within the five districts in the Free State Province namely: Thabo Mofutsanyana District in the Eastern Free State, Fezile Dabi and Lejweleputswa in the Northern Free State as well as Mangaung District, and Xhariep District in the Southern Free State Province. Following ethical approval, data was collected using a voice-recorder and field notes to collect data during focus group discussions. The participants were informed of the study purpose and a consent forms were signed. Data was collected from October to December 2017.

#### Inclusion criteria

1. All student accoucheurs registered at the Free State School of Nursing at their fourth year of training placed at post-natal units were purposively selected.
2. All student accoucheurs over the age of 18 years at their fourth year level of training who were willing to participate in the study and who signed the consent form were purposively selected.

#### Exclusion criteria

1. All student accoucheurs not studying at the Free State School of Nursing and not in their fourth-year level of their study and who are below the age of 18 years of age.
2. All student accoucheurs who meet the inclusion criteria but not willing to participate in the study.

### Focus group discussions

Five focus group discussions consisting of four to five members in a group were conducted. Additional two focus group discussions were added to ensure that data has been saturated. The focus group discussions were conducted with the fourth year student accoucheurs only based on the aim of the study which was to explore the experiences of student accoucheurs regarding their acceptance by pregnant women during their clinical placement at maternal healthcare in the Free State Province. Ground rules and information about the scope of discussion which assisted the participants to understand what is expected from them were laid ( Braun and Clarke, 2013 ). All focus group discussions were conducted in post-natal units' private room and lasted between 1 and 2 h. A grand tour guide question was posed followed by the probing open-ended questions. English was used as a media of instruction during focus group discussions. Data collection continued until data saturation was reached which was then analysed thematically ( Creswell and Plano Clark 2012 ).

### Demographic characteristics of the participants

As outlined in Table 1 , all study participants were black, unmarried males, between 20 and 24 years of age, in their fourth year level of training studying towards the Diploma in General Nurse (Community, Psychiatry) and Midwife (R.425) at the Free State School of Nursing.

### Data analysis

Qualitative data was transcribed from the voice-recorder into a written format, organised and stored using Atlas TI 8. version programme. This programme helped the researcher to systematically organised data into themes and sub-themes with codes to evaluate and interpret qualitative texts ( Creswell, 2014 ). Tesch's open-coding approach which includes reading, writing and making list of similar topics, abbreviating topics into codes and assembling data that belong to each category from which themes emerge.

### Research trustworthiness

Lincoln and Guba criteria of credibility, transferability, dependability and conformability were used to establish trustworthiness ( Lincoln and Guba, 1985 ). Conformability was accomplished by using bracketing and keeping a clear and easy-to-follow audit trail. Credibility was achieved through prolonged engagement with the data, sampling, data reviewing, codes, themes and sub-themes and member checking of the research participants. Dependability was checked by engaging two researchers who holds doctoral degrees to supervise the study. Participants who varied on demographic characteristics improved transferability.

### Ethical consideration

Prior to the commencement of the study, the approval of the study was obtained from the University Ethics Committee (IREC Number: 10/17) and the Free State Department of Health. Information letters detailing the purpose of the study were given to the participants. Confidentiality was assured, anonymity guaranteed and participants were allowed to withdraw from the study at any point. All participants gave informed consents agreeing to take part in the study.

## Findings

The analysed data was grouped into three categories of factors from which four major themes and six sub-themes emerged ( Table 2 ). These categories of factors affecting the quality of student accoucheurs' of training in maternal healthcare within the province were related to student nurses (student accoucheurs), maternal healthcare users (pregnant women) and nurse training/maternal healthcare service providers (Free State School of Nursing and Free State Department of Health hospitals). Table 2 represents the summary of categories, themes and sub-themes.

### Social interactions and relations

Most participants stated that the quality of their training in midwifery is based on good interactions with pregnant women. According to them, such interaction alleviates woman's preconceived ideas and fears about pregnancy, labour, delivery and the midwife that will be assisting them. Hence building a nurse–patient relationship was important in the maternity units, but this could be a challenge because most women are socialised into building sound relationships with other females. Therefore, the provision of quality training by the training institutions will empower them with knowledge, skills and support to build good nurse–patient relationships with pregnant women:

As males, we are challenged on how to build good relationships with women in maternity units. Most women come with preconceived ideas that they expect to be treated by female midwives that's how they were socialised […] (FG4, P3).

Most procedures are invasive such as vaginal examination […] and women refuses to be examined by us. I need help and support to interact and gain women's trust […] (FG2, P4).

Moreover, participants associated lack of communication skills as a contributing factor to women not accepting maternal healthcare rendered by them. Vernacular words used to give instructions to women during delivery could sound vulgar and embarrassing when are said by a male to a female:

Sometimes I feel shy to ask a woman to push in SeSotho as this word sound vulgar if used by male to a female […] (FG 4, P1).

I need to be taught how to give instructions to women without embarrassing them […] (FG5, P3).

### Transcultural diversity

Most participants stipulated that the training institutions should empower them with transcultural nursing in midwifery. Participants said women who are from traditional rural areas are religiously, socially and culturally sensitive regarding pregnancy as this is regarded as sacred to them hence, they become sceptical about maternal healthcare services rendered by males:

I was denied the opportunity by a Muslim family to assist during delivery as this was seen as taboo. I had to respect the patients' rights you know […] (FG5, P5).

Married women do not want to be seen naked by another man other than their own husbands. Despite this, culturally it is unacceptable to undress for a younger person of opposite gender […] (FG2, P3).

Some women will tell you that in their culture, men should wait for the period ten days before they can be allowed to see them during and after delivery […] (FG1, P1).

### Socio-economic status

It emanated from the participants that financially stable women are at liberty to choose the midwife of their preference, whether to attend private or public hospital for maternal healthcare services. Their voices in a public hospital regarding their choice of care is more respected. Therefore, they indicated that the training institutions should capacitate them with skills and knowledge in managing women attending their maternal healthcare services in the public hospitals irrespective of their financial statuses:

I have forfeited the chance of learning and fulfilling my required objectives other day when I was refused to deliver a lady teacher whose husband was a taxi owner […] and the Unit Manager did not intervene […] (FG5, P2).

Whereas financially unstable women are forced to attend public hospitals. Although some women do not express their views about student accoucheurs' presence in maternity units. Their resistance and non-co-operation can be detected when taking care of them which contribute to poor quality of their training in midwifery while increasing risks of maternal mortality:

Some women who are unemployed do not actually tell you that they do not want you as a male to assist them depriving us from quality training in midwifery, but they will resist your commands during examination and during labour putting themselves and the babies at risk […] (FG1, P5).

### Gender inequality in the work place

Student accoucheurs indicated that in clinical areas where they were allocated for maternal healthcare services in the province, no accoucheurs were placed in maternity units:

Accoucheurs are allocated in casualty, intensive care, theatre and other general wards except in maternity units […] (FG6, P4).

Most participants attested that even community health service accoucheurs were placed for the short period of time in this units. Therefore, women have limited exposure to maternal healthcare services rendered by accoucheurs; hence they reject and unaccepting to be taken care by them during their clinical placement in maternity units:

In some other few institutions you will find one or two community service accoucheurs working for either a week to a month […] and they are constantly taken to assist in other units if there is shortage of staff […] (FG6, P2).

Moreover, all Unit Managers, Assistant Director Nursing Managers responsible for maternal healthcare units were females including Lecturers teaching midwifery at the Free State School of Nursing. Although they render quality services in students training in midwifery, but gender diversity is not represented:

I have never saw even a single male Unit Manager or Assistant Director Nursing Manager where I was placed for my maternal healthcare rotation for the past two years […] and all our lecturers are females (FG7, P3).

Most student accoucheurs chose midwifery nursing science as their preferred discipline:

I like midwifery nursing […] and I prefer to work in maternity units rather than in other units […] (FG2, P5).

## Discussion

The study finding was based on the categories emerged during data analysis. The first category was based on student accoucheurs' related factors. The nurse training involves an active social interactions and relations with the patient. This enhances building a good nurse–patient relationship which promotes effective communication and provision of quality maternal healthcare to pregnant women. According to the Department of Health as cited by Mthombeni *et al.* (2018) mothers' needs during labour include physical comfort, emotional support and positive relationship provided by the attending midwife. Nurse–patient relationship is vital to gain patient's trust throughout the labour process ( Mthombeni *et al.* , 2018 ). It became evident from the participants' responses that they believed that pregnant women were socialised to interact with other women and form effective relations with each other. Kennedy *et al.* (2006) support the notion by stating that women-to-women relationship is characterised as nurturing, intuitive, patient, sensitive and understanding which is perceived by women as non-existent in student accoucheurs, hence most women refused maternal healthcare rendered by them. Therefore, it is the responsibility of midwives or student accoucheurs to build the relationship and inspire trust in pregnant women and their families during labour for the most positive pregnancy outcome ( Mthombeni *et al.* , 2018 ).

A gap was identified from the student accoucheurs discussion that they lack communication skills in assessing women and giving instructions effectively during delivery without causing discomfort to them and feeling embarrassed. The key facets of constructive communication in maternity care include an empathetic communication style, willingness to respond to questions and allowing enough time to discuss the woman's concerns ( Kozhimannil *et al.* , 2015 ). Student accoucheurs need to be supported and guided in building a good nurse–patient relationship including how to communicate effectively with women to gain their trust in maternity units. Therefore, it is imperative that the training institutions should empower student accoucheurs with efficient communication skills and building a sound nurse–patient relationship in maternal healthcare.

The maternal healthcare users' related factors were the second category emerged during data analysis. It emanated that women attending maternal healthcare where student accoucheurs are placed were from diverse cultural backgrounds which were religiously and culturally sensitive regarding pregnancy, labour and puerperium. Karout *et al.* (2013) concurred that a diverse culture differs in ethnicity, religion and language in which each group has its own value, belief system, tradition and different lifestyle. Hence it was taboo to most women to be seen naked, examined and delivered by student accoucheurs. Student accoucheurs affirmed that some Muslim women mentioned that it was against their religious belief to be assisted by a male during labour. Karout *et al.* (2013) reiterated that, according to Muslim religion, privacy was an important factor especially in the presence of mixed gender healthcare professionals. In the study conducted in Egypt, where most women are Muslims, male student midwives were also faced with rejection and lack of co-operation from pregnant women ( EL-Sayed and EL-Nemer, 2013 ). However, nearly all of the pregnant women preferred receiving treatment from male doctors during pregnancy and birth process instead of female doctors while they did not want to get care from student accoucheurs. Therefore, the training institutions should incorporate cultural diversity in teaching of student accoucheurs in midwifery care to ensure that student accoucheurs are equipped with knowledge to render maternal health to women of different cultures.

Whereas other student accoucheurs saw socio-economic status of women as a contributing factor towards their choice of maternal healthcare services preferred by them. The study conducted by Yaya *et al.* (2019) found that women with higher educational attainment and from higher wealth status households were more likely to deliver at healthcare facilities of their choice. Women and their families' socio-economic status such as occupation and income are the most important indicators for utilization of ANC, choice of delivery and types of assistance during delivery by women ( Haque, 2009 ). In most cases the sources of income are inadequate to meet the needs of the families ( Madlala *et al.* , 2018 ); hence other women with financial difficulties attend maternal healthcare services in the public institutions. This was confirmed by student accoucheurs who indicated that financial stable women rarely attend public health service for maternal healthcare services in comparison to financially unstable women. Although these women attend public health services during pregnancy, but they refuse maternal healthcare services rendered by student accoucheurs. This has a negative impact to the quality of training received by student accoucheurs in maternal healthcare. Inadequately trained accoucheurs poses a risk to already challenged maternal healthcare with high rates of maternal mortality.

The last category was based on factors related to the nurse training institutions and maternal healthcare institutions providing midwifery training to student accoucheurs. The Department of Health entrusted the maternal healthcare institutions and the Free State School of Nursing with the training of student nurses in midwifery discipline to capture the shortage of midwives and improving the quality of maternal healthcare services in the province. Student accoucheurs were concern with gender inequality in the work place at these institutions. Cude (2004) believes that if female nurses can be educated to act professionally when providing for the physical needs of male patients; therefore, the male nurse with the same education and dedication to the physical well-being of his patients can do likewise without violating the privacy and dignity of female patients. Furthermore, student accoucheurs argued that all midwives, midwifery lecturers and nursing managers working in maternity units were females. The lack of equal gender distribution in this institution deprives women of exposure to maternal healthcare services rendered by accoucheurs. Moreover, contribute to the rejection, resistance and discrimination against student accoucheurs by women leading to poorly trained accoucheurs to render quality maternal healthcare services on completion of their training. Folami (2017) strongly criticised this practice and suggested that nursing institutions should ensure that gender bias and stereotypes are minimised to provide equitable learning situations for student accoucheurs as well as creating a nursing working force that reflects greater gender diversity. Furthermore, most student accoucheurs expressed their passion in midwifery. Student accoucheurs suggested that stakeholders involved in maternal healthcare services should consider accoucheurs' work discipline preferences within their institutions.

### Development of guidelines to facilitate acceptance of student accoucheurs in maternal healthcare clinical practice and improve quality of their training in midwifery

The guidelines were developed to realise the aim of the study. Furthermore, these guidelines were developed and recommended for the following purposes: for implementation with the aim to inform/guide the stakeholders involved in the training of student accoucheurs regarding possible ways to facilitate acceptance of student accoucheurs in clinical practice in maternal healthcare institutions; to improve the quality of training student accoucheurs in maternal healthcare; to target policymakers in the Free State Department of Health, the Nurse Managers in the maternal healthcare institutions and the nurse training institutions in the province. The development of these guidelines was guided by the Nominal Group Technique (NGT). The NGT gathers information by asking the participants to respond to questions posed by the researcher and then asking the participants to prioritise the ideas of all group members ( Evaluation Briefs, 2006 ). Table 3 represents the three sets of developed guidelines.

#### A1. Integration of cultural and religious sensitivity in the provision of maternal healthcare services in maternal healthcare institutions

The policymakers in the Free State Department of Health need to ensure that maternal healthcare services rendered at the maternal health institutions cater for all pregnant women despite their cultural or religious backgrounds, whilst ensuring effective and quality training of student accoucheurs in midwifery. Therefore, the following recommendations for the implementation of this guideline should be taken into consideration:

1. A policy which incorporates cultural or religious sensitivity of pregnant women to student accoucheurs' presence in the maternal healthcare institutions should be developed and implemented.
2. A policy that integrates cultural and religious sensitivity in maternal healthcare in the curriculum of the Free State School of Nursing to ensure that student accoucheurs are taught about pregnant women's religious and cultural diversity.

#### B1. Distribution of accoucheurs in the maternal healthcare institutions accredited by SANC for student accoucheurs' training in midwifery

The Nurse Managers places accoucheurs in all other nursing units within the organisations except in the maternal healthcare units' despite being trained to work in this discipline. This deprives pregnant women an opportunity to experience the maternal healthcare services rendered by accoucheurs. Hence the recommendations for implementation of the developed guideline by Nurse Managers.

1. Nurse Managers should allocate accoucheurs on a rotational basis in the maternal healthcare units to improve the visibility of accoucheurs in these units.
2. Nurse Managers should consider accoucheurs when vacant positions for Unit Managers in maternal healthcare become available to ensure gender equity in the work place.
3. Placement of accoucheurs in maternal healthcare units will improve the quality of student accoucheurs' training by providing support and encouragement in midwifery discipline.

#### B2. Accoucheurs' freedom to exercise their right to choose preferred disciplines in the healthcare institutions

Nursing Managers need to consider how accoucheurs are allocated in the Free State healthcare institutions. The random allocation of accoucheurs in general units except for maternity units, despite their preference, deprives them of the opportunity to explore and grow their careers in maternal healthcare. It is recommended that the developed guideline should be implemented by Nurse Managers:

1. Nurse Managers should ascertain accoucheurs' preferred discipline and negotiate placement on a rotational basis with them to promote gender equity in maternal healthcare institutions.
2. Accoucheurs requesting to be placed in maternal healthcare units should be granted the opportunity to practice midwifery.
3. Accoucheurs who have an interest in improving their career by furthering their studies in advanced midwifery should be given the relevant opportunities by Nurse Managers.
4. Accoucheurs should be given opportunities to mentor and supervise student accoucheurs in the maternal healthcare institutions to improve the quality of their training in midwifery discipline.

#### C1. Development and implementation of a teaching programme for pregnant women on gender diversity and equity in nursing

Student accoucheurs undergo the same training as their female counterparts' despite being rejected and discriminated by women in maternal healthcare units due to their gender. It is recommended that a career exposure programme that will focus on teaching pregnant women and their family members about career choice and the contribution of student accoucheurs in maternal healthcare is required. Hence the researcher made recommendations to implement the developed guideline:

1. A programme designed to teach pregnant women in ANC, labour wards and postnatal wards about gender and its importance in nursing, and to equip pregnant women and their families with knowledge about the nursing profession.
2. Involve the relevant training stakeholders in the implementation of the teaching programme about gender diversity and equity in nursing to ensure correct implementation of the programme at the Free State maternal healthcare institutions.
3. Involve student accoucheurs in teaching pregnant women and their families about their roles in maternal healthcare to promote gender diversity and equity in nursing.
4. Encourage pregnant women to verbalise their concerns and uncertainties about student accoucheurs in maternal healthcare and to give clear and accurate responses.

#### C2. Development and implementation of a teaching programme on communication skills for student accoucheurs in maternal healthcare

The development of a programme that will integrate communication skills specifically in maternal healthcare for student accoucheurs in their third- and fourth-year level of training is necessary. This programme will facilitate effective communication skills for student accoucheurs as the key facets of constructive communication in maternity units ( Kozhimannil *et al.* , 2015 ). The following are recommendations for the implementation of the developed guideline:

1. Lecturers at the Free State School of Nursing should develop and integrate communication skills in their teaching objectives to equip student accoucheurs with effective communication skills in maternal healthcare.
2. Translation of culturally sensitive terminologies, used in midwifery, into a less embarrassing and non-offensive form so that student accoucheurs and pregnant women are not embarrassed.
3. Lecturers should encourage student accoucheurs to implement the taught communications skills, acquired in their training, during their clinical placement at the Free State maternal healthcare institutions.
4. Student accoucheurs should give feedback regarding the effectiveness of implemented communication skills programme to lecturers on a regular basis.

#### C3. Clinical instructors stationed at the Free State maternal healthcare institutions

The Free State School of Nursing should consider hiring clinical instructors to be stationed in the maternity units. The developed guideline and the proposed implementation is aimed at improving the supervision of student accoucheurs by clinical instructors stationed at the Free State maternal healthcare institutions. It is imperative that the Free State School of Nursing consider the recommendations below for implementing the developed guideline:

1. Create, advertise and approve posts with specific requirements for clinical instructors while considering gender diversity and equity to be allocated in various maternal healthcare institutions in the Free State Province, employed by the Free State School of Nursing.
2. Enrolment of clinical instructors in a simulation course for health professionals to equip them with needed skills and knowledge on clinical accompaniment of students including student accoucheurs to improve the quality of training in maternal healthcare.
3. Allocation of clinical instructors in the Free State maternal healthcare institutions accredited for placement of student accoucheurs in midwifery discipline to ensure prompt and active supervision of student accoucheurs to improve the quality of their training.
4. Clinical instructors should actively utilise the simulation skills laboratory at the Free State School of Nursing in demonstrating skills and feedback from student accoucheurs to ensure quality of training in midwifery discipline.
5. Clinical instructors at the Free State maternal healthcare institutions should frequently give feedback to lecturers about challenges, progress and proposed solutions regarding student accoucheurs placed in these institutions.

#### C4. Utilisation of upgraded skills laboratory with high and low fidelity manikins for midwifery simulation of clinical skills at the Free State School of nursing

The Free State School of Nursing should design and equip clinical skills laboratory with the high and low fidelity manikins designed for midwifery teaching purposes. This skills laboratory should be fully utilised for midwifery clinical practice to enhance skills and knowledge of student midwives and student accoucheurs enabling them to render quality maternal healthcare services. The Free State School of Nursing academia should consider the following recommendations for implementation of this guideline:

1. A designed programme with stipulated time frames for clinical skills demonstrations and feedback for student accoucheurs to improve the quality of training in maternal healthcare.
2. Compulsory attendance and supervision of student accoucheurs during clinical practice in the skills laboratories.
3. Use simulated patients in the skills laboratory for maternal healthcare history taking to teach student accoucheurs effective communication skills improve their confidence in maternal healthcare.
4. Giving constructive feedback, either negative or positive to student accoucheurs after the performance of their clinical tasks to encourage them to improve their skills and knowledge.
5. Debriefing sessions after each clinical task should be conducted to ensure that student accoucheurs express their fears and concerns which should be addressed by clinical instructors.
6. Counselling sessions and referrals to student counsellors those student accoucheurs with difficulty in maternal healthcare clinical practice in the skills laboratory.
7. Compulsory briefing sessions by lecturers for student accoucheurs on what to expect and how to deal with rejection by pregnant women prior to their clinical placement in the Free State maternal healthcare institutions.

## Limitations

The study was conducted in the Free State Province maternal healthcare institutions accredited by SANC for student nurse training purposes which may restrict transferability. Lincoln and Guba (1985) state that transferability refers to the applicability of findings to other settings. The participants purposely selected for the study were student accoucheurs in their fourth-year level of training studying at the Free State School of Nursing. Therefore, the study could have yielded different findings if the student accoucheurs from the Free State University, academia from Free State School of Nursing, policymakers at the Free State Department of Health, accoucheurs and Nurse Managers were included in the sample.

## Implications of the research

The study is in line with nursing education in the midwifery discipline and human resource management. The developed guidelines are intended for implementation by the policymakers in the Free State Department of Health, Free State School of Nursing and the Nurse Managers in their respective maternal healthcare institutions in the Free State Province including to other relevant maternal healthcare institutions involved in midwifery student nurse training. The researcher contends that this is the first comprehensive study that has researched and developed guidelines regarding the phenomena. According to Thomas (2017) , developed guidelines should be evaluated by various methods such as asking the professionals not involved in the guidelines development process to review them for clarity, internal consistency and acceptability. Therefore, it is envisaged that the developed guidelines be disseminated to the wider healthcare communities for its evaluation, recommendations and implementation. The study is a contribution to the emerging education and training of student accoucheurs body of knowledge in the Free State Province and to the global existing body of knowledge regarding the phenomena.

## Conclusion

The literature review and data analysis from the study participants confirmed that there were gaps in the training of student accoucheurs and that there were no developed guidelines with respect to improving the quality of training and support of student accoucheurs during their clinical placement in maternal healthcare institutions. The main aim of the current study was realised in that the guidelines to facilitate acceptance and improve the quality of training of student accoucheurs in midwifery clinical practice at the Free State maternal healthcare institutions were developed. Recommendations for implementation of the developed guidelines were suggested for stakeholders involved in the training of student accoucheurs in midwifery discipline.

## Figures and Tables

**Table 1:** Represent the demographic information of the study participants

Age	Race	Level of training	Course	Total
20–24	Black	4th year	Diploma in General Nurse (Community, Psychiatry) and Midwife (R.425)	32

**Table 2:** Summary of categories, themes and sub-themes

Categories	Themes	Sub-Themes
Student accoucheurs related factors	Social interactions and relations	Nurse–patient relationship
		Verbal communication
Maternal healthcare users-related factors	Transcultural diversity	Cultural beliefs, values and care during pregnancy, labour and puerperium in the maternal health
	Socio-economic status	Financial stability of women
Nurse training institutions and maternal healthcare service providers' related factors	Gender inequality in the work place	Human resource gender distribution in the workplace
		Accoucheurs' work discipline preference

**Table 3:** Guidelines for student accoucheurs' acceptance in maternal healthcare

Guideline	Targeted stakeholders	Focus area
Guideline A	Guideline for policymakers in the Free State Department of Health	A1. Integration of cultural and religious sensitivity in the provision of maternal healthcare services in maternal healthcare institutions
Guideline B	Guidelines for Nurse Managers in the maternal healthcare institutions	B1. Distribution of accoucheurs in the maternal healthcare institutions
		B2. Accoucheurs' freedom to exercise their right to choose preferred disciplines in the healthcare institutions
Guideline C	Guidelines for the Free State School of Nursing	C1. Development and implementation of a teaching programme for pregnant women on gender diversity and equity in nursing
		C2. Development and implementation of a teaching programme on communication skills for student accoucheurs in maternal healthcare
		C3. Clinical instructors stationed at the Free State maternal healthcare institutions
		C4. Utilisation of upgraded skills laboratory with high and low fidelity manikins for midwifery simulation of clinical skills at the Free State School of Nursing
